# Medication Use and Storage, and Their Potential Risks in US Households

**DOI:** 10.3390/pharmacy10010027

**Published:** 2022-02-09

**Authors:** SuHak Lee, Jon C. Schommer

**Affiliations:** College of Pharmacy, University of Minnesota, 308 Harvard Street, S.E., Minneapolis, MN 55455, USA; schom010@umn.edu

**Keywords:** medication, storage, risk, reuse, household, inventory

## Abstract

Background: Medications stored in US households may pose risks to vulnerable populations and the environment, potentially increasing societal costs. Research regarding these aspects is scant, and interventions like medication reuse may alleviate negative consequences. The purpose of this study was to describe medications stored in US households, gauge their potential risk to minors (under 18 years of age), pets, and the environment, and estimate potential costs of unused medications. Methods: A survey of 220 US Qualtrics panel members was completed regarding medications stored at home. Published literature guided data coding for risks to minors, pets, and the environment and for estimating potential costs of unused medications. Results: Of the 192 households who provided usable and complete data, 154 (80%) reported storing a medication at home. Most medications were taken daily for chronic diseases. The majority of households with residents or guests who are minors and those with pets reported storing medications with a high risk of poisoning in easily accessible areas such as counters. Regarding risk to the aquatic environment, 46% of the medications had published data regarding this risk. For those with published data, 42% presented a level of significant risk to the aquatic environment. Unused medications stored at home had an estimated potential cost of $98 million at a national level. Implications/Conclusions: Medications stored at home may pose risks to vulnerable populations and the environment. More research regarding medications stored in households and their risks is required to develop innovative interventions such as medication reuse to prevent any potential harm.

## 1. Introduction

With the increasing use of prescription and over-the-counter (OTC) medications, more drug products are being accumulated in US households [1,2,3,4,5,6,7,8,9,10,11,12,13,14]. Larger medication inventories at home, and subsequent waste can endanger patient safety, reduce quality of care, and harm the environment. To develop interventions that efficiently mitigate unintended, negative consequences, there is a need to study medication use, storage, and disposal in households more in-depth and comprehensively. For instance, medication reuse pertains to redispensing of medications that were once acquired by an individual or healthcare facility. Redistributing unused medications can reduce healthcare waste and costs, and enhance access to care [15,16,17,18,19,20,21,22]. Patients are recognized as the primary consumers of reused medications and as one of the potential primary sources of medications to be reused [23,24,25,26,27]. Therefore, understanding the interplay between patients and their medications will clarify the types of risk that medication reuse can minimize, guide its efficient implementation, and illuminate its benefit. However, comprehensive research regarding the use, storage and disposal of their medications especially in the US is scant.

Accumulating medication inventories at home can harm patients and their families by increasing the risk of medication poisoning. Sorensen et al., found that the higher number of medications stored at home may increase the risk of taking someone else’s medications within the same household [28]. According to the 2019 report of the American Association of Poison Control Center’s National Poison Data System (NPDS), out of over two million reported exposure cases, 92.1% occurred in residence either of their own or someone else’s. The poisoning of patients younger than 20 years of age comprised 57.5% of the reported exposure cases, so they seem to be particularly at higher risk than other age groups [29]. The NPDS reports from the previous years showed similar trends [30,31,32,33]. In addition to the high rate of occurrence, poisoning accidents of minors can cause injuries leading to emergency department visits and at times be fatal, but they are preventable and should be critically discussed [29,30,31,32,33,34,35,36].

Selective serotonin reuptake inhibitors (SSRIs), nonsteroidal inflammatory drugs (NSAIDs), acetaminophen (APAP), histamine-1 receptor antagonists (H1RAs), and sedatives/hypnotics/antipsychotics (SHAs) have been identified as medications commonly involved in child poisoning in the NPDS reports [12,13,14,28,29]. Opioids have also been identified as harmful and high-risk medications for poisoning of minors [35,36,37,38,39,40,41,42,43,44,45]. Regardless of the types of medications, the ease of access seems to play a significant role in pediatric exposure. For example, one survey that analyzed children who were poisoned by their grandparents’ medications found medications stored in easily accessible locations such as shelves lower than three feet from the floor were significantly more involved in poisoning than those stored in high shelves [34]. Storing in closed spaces like drawers and closets would also provide additional physical barriers and keep medications away from children more effectively. Nevertheless, for opioids which are extremely habit forming, two studies found that 26% and 36% of the participants stored them in open spaces at home, respectively, noting unsafe storage of the high-risk medication [37,38].

The NPDS report also showed that 98.6% of all non-human exposures involved dogs or cats, implying that these household pets may be at risk of poisoning [29]. The NPDS reports did not specify the substances involved in these cases, but Cortinovis et al. comprehensively reviewed the drugs intended for human use that were frequently involved in poisoning of dogs and cats. Most medications of concern in the review were the same as the high-risk medications for humans, while some, such as vitamin D, iron salts, and β2-agonists seem to be high-risk more specifically for dogs and cats [46].

In addition to poisoning, accumulation of unused, unwanted, and expired (UUE) medications at home in the US has been frequently reported in the literature [13,14,37,39,40,41,42,43]. The accumulation of UUE medications may represent inefficient medication utilization and a potential source of financial waste in healthcare. The economical loss may not seem so apparent, as no significant difference in total prescription costs between those who had any unused medications and who did not was found [14]. However, these medications are stored without fulfilling their intended consumption goals. They can continuously require storage costs and hamper adequate access to medications for other potential purchasers that could have benefited from their use [47,48].

It is concerning when UUE medications are discarded in the end, especially because the most common locations of medication disposal were identified as garbage, toilet and sink in the literature [11,38,39,42]. These disposal methods are also recommended by the Food and Drug Administration (FDA) [49]. However, with these methods, pharmaceuticals still can be introduced into the water system and eventually into the groundwater, lakes, and streams, harming the environment and potentially humans [12,22,27,50,51,52]. Considering the negative implications, assessment of the potential environmental effects of medications stored at home is imperative. Such an assessment would reaffirm the significance of the environmental issues associated with these medications and help develop better disposal practices to minimize environmental harm.

The 2014–15 Environmentally Classified Pharmaceuticals report by the Stockholm County Council provides the most comprehensive assessment of various medications’ environmental effects [53]. However, the evidence provided by the report was based on the Swedish water system and their standard medication doses, and may not be fully applicable in the US. Despite the shortcomings, no study has critically explored the potential environmental risk of medications stored in US households. The Stockholm report can serve as a foundational reference for exploration and basic assessment of the potential risk.

Besides the different types of risk discussed above, the higher number of medications stored at home has been associated with deeper underlying issues with patients such as high severity of illness, therapeutic duplication, confusion between generic and trade names, low medication adherence and lack of medication administration routine [28]. Possessing unused medications also has been associated with a greater number of comorbidities, more frequent visits to emergency departments, primary care physicians, or specialists, and higher total medical cost of care [14]. Similar to these factors, polypharmacy, commonly defined as concurrent use of five or more medications, seems to be strongly associated with greater and unnecessary medication use [54]. The older population especially has a higher chance of comorbidity and is more likely to experience polypharmacy. Maneuvering through multiple, intricate medication therapies can be burdensome for many [54,55,56,57,58]. For this reason, when older patients manage their medications on their own, polypharmacy can arguably contribute to low medication adherence [59,60,61], potentially creating an unnecessary reservoir of medications stored at home.

Research assessing the aforementioned risks and economic implications of medications stored in the US households is scant. To fill the gaps in the literature, the first objective of the study was to describe medications stored in U.S. households including the number, indications, frequency of use, and storage locations. The second objective was to evaluate unintended consequences of these medications regarding (a) risk for poisoning of minors, (b) risk for poisoning of pets, and (c) risk to the environment. The third objective was to estimate the potential economic cost of the unused medications stored at home.

## 2. Materials and Methods

**Figure 1:**
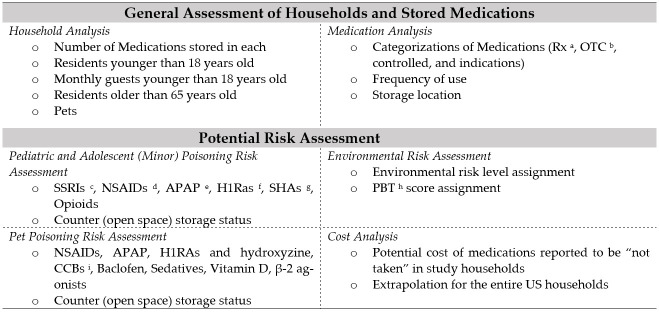
Study Overview (^a^: prescription only, ^b^: over-the-counter, ^c^: serotonin reuptake inhibitors, ^d^: nonsteroidal inflammatory drugs, ^e^: acetaminophen, ^f^: histamine-1 receptor antagonists, ^g^: sedative/hypnotics/antipsychotics, ^h^: persistence (P), bioaccumulation (B), and toxicity (T), ^i^: calcium channel blockers).

The 2018 National Household Medication Inventory Survey was the data source for this cross-sectional study. The survey was deemed to be non-human research and exempt from full review by the University of Minnesota Institutional Review Board. A total of 220 Qualtrics panel members in the U.S. were surveyed from May–June 2018. The Qualtrics Panel members who volunteered to participate in the survey received an invitation from Qualtrics, and the survey was self-administered. Upon completing the survey, each panel member earned credits which were reimbursed monetarily later. The overview of the data analysis is shown in Figure 1.

### 2.1. General Assessment of Households and Stored Medications

#### 2.1.1. Household Analysis

In the survey, the participants were asked to choose from “0 medication,” “1–4 medication(s),” “5–10 medications,” or “more than 10 medications” for the number of medications stored by each household. The participants who reported storing no medication were asked to stop at the beginning of the survey without answering any subsequent questions about the household members.

The survey also assessed whether a household had a resident under 18 years old, a monthly guest under 18 years old, a resident older than 65 years old, and a pet. The Fisher’s exact test was utilized to compare the number of medications stored by the households with at least one resident older than 65 years and those without.

#### 2.1.2. Medication Analysis

(a) Categorization of medications

The names of medications the participants stored in their households were reviewed and categorized by their prescription status (prescription, controlled substance, or OTC) and common indications. The controlled substance status was determined based on the Controlled Substances Act, following the federal classification. Medications like aspirin and omeprazole which can be available both as prescription and OTC, were categorized as OTC.

The typical indications of the reported medications were determined by the principal investigator (S.L.) who practices as a pharmacist in Minnesota, USA. The categorization of indications intended to be as inclusive as possible without having much overlap among the indications. A detailed description of the process of assigning medication indications is provided in Appendix A. A response with a typo that hindered interpretation of the exact name of the medication was categorized as “invalid.” When the same medication was reported more than once by the same household, any responses reported subsequently to the first response were categorized as “duplicate.”

(b) Medication frequency of use and storage locations

For the frequency of use of each medication, the participants were asked to choose from “taken daily,” “taken as needed,” “not taken, saving for future,” “not taken, would like to discard,” and “other.” The participants were not given an option to specify “other.” For the storage location of each medication, they were asked to choose from “bathroom counter,” “bathroom cabinet,” “garage,” “kitchen counter,” “kitchen cabinet or drawer,” “utility room,” “hallway closet,” “bedroom counter,” “bedroom cabinet,” “bedroom closet,” and “other.” The participants were not asked to specify “other” in the survey.

### 2.2. Potential Risk of Poisoning Analysis

The risk analysis assessed whether high-risk medications for poisoning of minors and pets were stored on the counter by the households with a resident or monthly guest younger than 18 years old and a pet. Based on the literature, high-risk medications were determined as those more commonly involved in poisoning or associated with serious poisoning with harmful outcomes for minors and pets, particularly dogs and cats. The types of pets owned by the households were not asked in the survey, and it was assumed that the households owned either dogs or cats for simplicity and to adapt the findings of Cortinovis et al. [46].

The high-risk medications for minors included selective serotonin reuptake inhibitors (SSRIs), nonsteroidal inflammatory drugs (NSAIDs), acetaminophen (APAP), histamine-1 receptor antagonists (H1RAs), sedative/hypnotics/antipsychotics (SHAs), and opioids [29,30,31,32,33]. In the National Poison Data System (NPDS) reports, the SHA medications are comprised of barbiturates, atypical antipsychotics, benzodiazepines, buspirone, chloral hydrate, ethchlorvynol, meprobamate, methaqualone, phenothiazines, and histamine-related OTC sleep aids excluding diphenhydramine [29,30,31,32,33]. The high-risk medications for dogs and cats included analgesics (NSAIDs and acetaminophen), antihistamines (diphenhydramine, doxylamine, hydroxyzine, loratadine), calcium channel blockers (CCBs), SSRIs, baclofen, sedative-hypnotic drugs such as benzodiazepines, and non-benzodiazepine hypnotic sedatives, loperamide, vitamin D, and β2-adrenergic receptor agonists [46].

### 2.3. Potential Environmental Risk Analysis

Based on the 2014–15 Environmentally Classified Pharmaceuticals published by the Stockholm County Council, each reported medication was assigned with a risk of toxicity to the aquatic environment and Persistence, Bioaccumulation, Toxicity (PBT) score. The persistence (P), bioaccumulation (B), and toxicity (T) of the PBT scores represent the ability to resist degradation in the aquatic environment, accumulation in adipose tissues of aquatic organisms, and the potential to poison aquatic organisms, respectively. Each characteristic is assigned a score ranging from 0–3, with a higher value indicating a higher risk. The sums of the scores of the three characteristics of medications have been reported as the PBD Index and utilized for the analysis in the current study [53].

The risk levels were classified as “insignificant,” “low,” “moderate,” and “high.” Medications that had undetermined risk levels due to insufficient evidence or were not mentioned in the report were categorized as “insufficient data.” Vitamins, electrolytes, amino acids, peptides, proteins, carbohydrates, lipids, vaccines, and herbal medicine were not considered to pose a risk to the environment in the Stockholm report and were given the “exempt” status [53].

For combination medications whose active ingredients could be identified with the given response, the highest known risk level and highest known PBT score of the comprising ingredients were assigned. For example, when the comprising ingredients had both “insufficient data” and “insignificant” risk levels, the “insignificant” ingredient was determined to have more conclusive evidence for the risk and deemed the higher known risk level.

### 2.4. Cost Analysis

The potential cost of the medications that were reported to be either “not taken, saving for future” or “not taken, would like to discard” was assessed. The survey did not specify the units for quantities and strengths of medications to be reported for the participants. Without standardized units, the responses for quantities and strengths did not show a particular trend and could not be used for cost analysis. In order to estimate the potential cost, the sum of the lowest package Average Wholesale Price (AWP) on Red Book^®^ for each medication regardless of the dosage form, strength, and package size was utilized [62]. The sum was then extrapolated to a national level, based on the US census data [63]. Utilizing the lowest unit AWP was considered, but it was suspected that the chance of storing multiple units of a medication would be higher than storing just one unit. Therefore, the next lowest cost estimate available which was the lowest package AWP at the time of the analysis in 2021 was utilized for the analysis.

Once the total potential cost of “not taken” medications was determined, the ratio of the number of US households based on the US census data (120,756,048 households) [63] and the number of households storing those medications was used to extrapolate the cost nationally.

The survey results were analyzed with Microsoft Excel 2016, SPSS (v. 27.0), and R (v.4.1.0).

## 3. Results

### 3.1. General Assessment of Households and Stored Medications

#### 3.1.1. Household Analysis

A total of 192 households (87.3%) out of the 220 households who volunteered to participate completed the survey. The zip codes of the participating households matched the geographic distribution of the US census data, indicating that the collected data were nationally representative [63]. The number of medications stored in the households is shown in Table 1. Note that 154 households (80.2%) reported storing at least one medication at home.

Forty-six households (24%) had at least one resident older than 65 years old (Table 1). The Fisher’s exact test determined no significant difference in the number of medications reported by the households with a resident older than 65 years and the number reported by those without (*p* = 0.10).

#### 3.1.2. Medication Analysis

(a) Categorization of medications

A total of 457 medications stored at home were reported. After excluding eight “invalid” and 45 “duplicate” responses, a total of 404 valid responses were included in the analysis. Of the valid responses, 261 medications (64.6%) were prescription-only and 143 medications (35.4%) were OTC. Among the prescription-only medications, 25 medications (9.6%) were controlled substances. Table 2 has the breakdown of the indications of the reported prescription, controlled, and OTC medications. The three most commonly reported indications for prescription-only medications were cardiovascular therapy (33.5%), mental health therapy (18.6%), and endocrine therapy (16.5%). Mental health conditions (60%) such as attention deficit hyperactivity disorder (ADHD), and anxiety were the most commonly reported indications for controlled substances. The three most commonly reported indications for OTC medications were pain (37.1%), supplements (18.2%), and gastrointestinal therapy (13.3%) (Table 2). Among the households storing at least one medication at home, 72 households (46.7%) had at least one OTC medication stored at home. The crude responses for medication names are categorized by indications in Appendix B, Appendix C, Appendix D and Appendix E.

(b) Medication frequency of use and storage locations

Some of the responses for medication names categorized as “invalid” had their valid frequencies and locations reported. Also, a majority of the medications categorized as “duplicate” had different storage locations. For comprehensiveness, the frequency and location responses corresponding to “duplicate” or “invalid” in the medication indication analysis were included in the current analysis. The inclusion of these responses in the analysis yielded a total number of samples higher than the number of medications reported in the categorization.

A total of 465 responses for the frequency of use was collected. Table 3 shows most of the reported medications were being used: “taken daily,” and “taken as needed” (93.8%).

**Figure 2:**
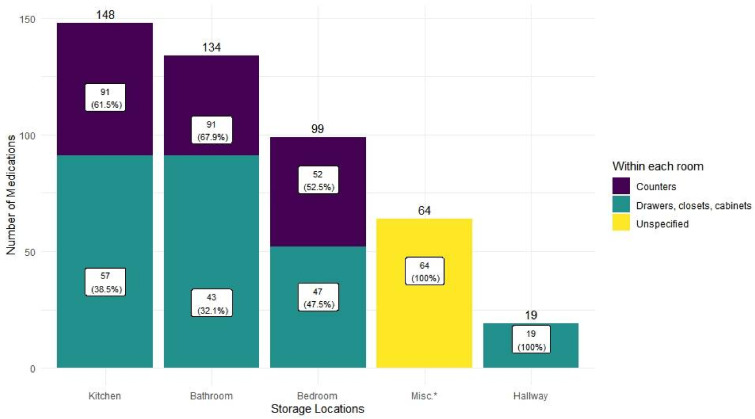
Medication storage locations (*n* = 464) * Misc. in the x-axis includes 14 “utility room,” 8 “garage,” and 42 “other.” The participants were not asked to specify “other” in the survey.

For storage locations, a total of 464 responses was collected. Most medications were stored in kitchens (31.9%), bathrooms (28.9%), and bedrooms (21.3%). A total of 147 medications (31.7%) were stored on open counters in bathrooms, kitchens, or bedrooms, which would be more accessible than those stored in drawers, closets, or cabinets (Figure 2). Two households submitted different numbers of responses for the frequencies and locations for their medications, and yielded different sample sizes (*n* = 465 vs. *n* = 464).

### 3.2. Potential Risk of Poisoning Analysis

Among households storing at least one medication (*n* = 154), 75 (39.1%) had at least one resident younger than 18 years old, 55 (28.6%) had at least one monthly guest younger than 18 years old, and 112 (58.3%) had at least one pet.

A total of five out of the six (83%) high-risk medications (all except opioids) was being stored on the counter by at least one household with one or more resident(s) younger than 18 years old. At least one household with one or more monthly guest(s) younger than 18 years old stored four out of the six (67%) high-risk medications (all except selective serotonin reuptake inhibitors (SSRIs) and opioids) on the counter. Of the nine high-risk medications, seven (78%) (all except vitamin D and baclofen) were being stored on the counter by at least one household with one or more pet(s). In fact, baclofen storage was not reported by any households with one or more pets.

### 3.3. Potential Environmental Risk Analysis

After excluding “duplicate” and “invalid” responses, a total of 404 valid medications reported in the survey were included in the environmental analysis and reviewed. Of the valid responses, six OTC medications had only their brand names reported, and were excluded from the current analyses. These brand medications are available in different variations of active ingredients, but the specific types were not reported in the survey. A total of 27 medications were “exempt” from the risk analysis per the 2014–15 Environmentally Classified Pharmaceuticals by the Stockholm County Council [53].

**Figure 3:**
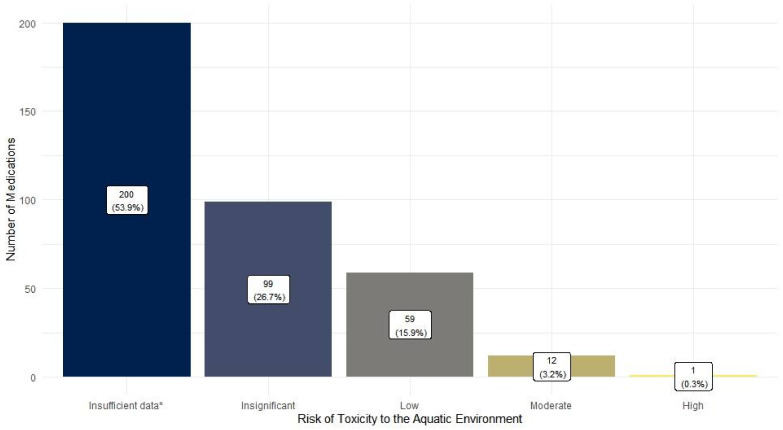
Toxic risk levels assigned to the aquatic environment of the reported medications based on the 2014–15 Environmentally Classified Pharmaceuticals by the Stockholm County Council [53] (*n* = 371) * “Insufficient data” includes medications with undetermined risk levels due to insufficient evidence or those that were not mentioned in the Stockholm report.

**Figure 4:**
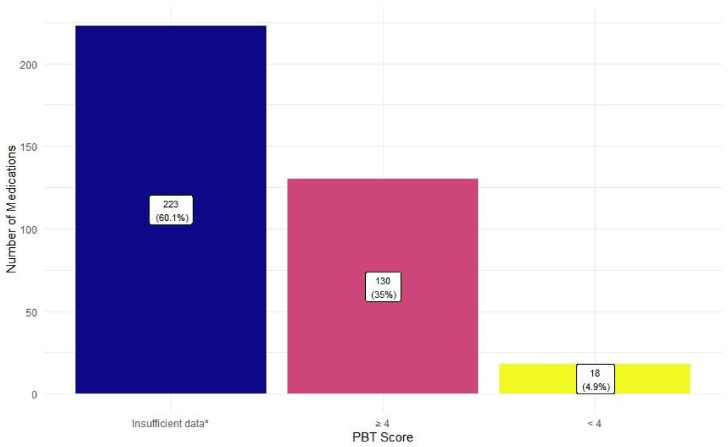
Persistence (P), Bioaccumulation (B), and Toxicity (T) scores of the reported medications. The PBT score is a sum of the P, B, and T score, each ranging from 0–3, assigned to a particular medication reported in the 2014–15 Environmentally Classified Pharmaceuticals by the Stockholm County Council. The higher the score, the higher the risk [53] (*n* = 371) * Medications without a PBT score in the Stockholm report were categorized as “insufficient data.”.

A majority of the medications, 53.9% and 60.1% of the medications did not have sufficient data to determine their risk of toxicity to the aquatic environment and their Persistence (P), Bioaccumulation (B), Toxicity (T) scores respectively (Figure 3 and Figure 4). Among those with data, medications with insignificant-risk level (26.7%) were most prevalent (Figure 3). On the other hand, medications with PBT scores of 4 or higher (35%) were far more frequently identified compared to those with PBT scores lower than 4 (4.9%) (Figure 4).

### 3.4. Cost Analysis

Out of the 19 “not taken, saving for future” or “not taken, would like to discard” responses, 14 had appropriately reported medication names. Based on the lowest package AWP, the 14 medications were worth $156.54. Extrapolating this result to a national level, $98,453,915.39 of medications were potentially stored at home without being used and potentially being wasted.

## 4. Discussion

### 4.1. Objective #1: To Assess the US Household Members and the Number, Indications, Frequency of Use and Storage Locations of Their Medications Stored at Home

Approximately 20% of the participating households did not store any medications at home. On the other hand, a household survey conducted in IL found all participating households storing at least one prescription or OTC medication at home [11]. No other US household surveys that could be used as a reference were identified during the literature review. Other similar studies assessed medication possession by individuals, not households.

The bivariate comparison of the number of medications between the households with and without any residents older than 65 found no statistical difference. However, the current survey did not collect the number of medications specifically stored by the individual residents older than 65, and the statistical analysis was explorative at best. The self-administered and online nature of the survey may have also heightened the barrier for the elderly to actively participate in the household survey.

The three most prevalent indications of the reported prescription medications were cardiovascular therapy, mental health, and endocrine therapy including diabetes. The Centers for Disease Control and Prevention (CDC) reported that 6 in 10 US adults suffer from chronic diseases including but not limited to the three identified in the current study. The most common indication of the reported OTC medications was “pain.” This result may correspond to arthritis, another prevalent chronic disease reported by the CDC and often managed with analgesics. The prevalence of chronic diseases was also reflected in the most common medication frequency of use being “taken daily” [64,65,66,67].

Approximately a half of the households reported storing at least one OTC medication at home, consistent with the high prevalence of OTC medication use published in the literature. 8 of 10 US patients do not seek help from a healthcare professional initially for their minor illnesses and resort to OTC medications [9]. Considering the high barrier to healthcare access in the US, OTC treatment can be a convenient option for many patients.

Most medications reported in the study were stored in bedrooms, kitchen, and bathrooms. The alarming trend was a high number of medications being stored in bathrooms, which is inappropriate for medication storage. Funk et al. did a separate analysis for the appropriateness for each reported medication and their storage space, utilizing the published humidity and temperature ranges of various household locations and specific medication storage recommendations [68].

### 4.2. Objective #2: To Evaluate the Potential Risk for Poisoning of Minors and Pets and for the Environment Posed by the Medications Stored in the Study Households

#### 4.2.1. Poisoning Risk

A considerable amount (37.1%) of the reported medications were being stored on open counters in kitchens, bathrooms, and bedrooms. In addition, most of the high-risk medications for pediatric and adolescent poisoning were stored on counters by at least one household with a minor or pet. Counters are easily accessible and are not appropriate for medication storage, especially for households with vulnerable populations. In order to prevent and minimize harm by pediatric medication poisoning at home, the CDC recommends storing medications up and away and out of sight in a cabinet where a child cannot reach, never leaving medications unattended when a child is around, and having the Poison Help number readily available in the household [69]. It is uncertain whether patients living with minors or frequently having minor guests are educated about the importance of storage locations and how appropriately they store medications to prevent poisoning. As for pets, although only a small number of calcium channel blockers were reported, they have a small margin of safety, and ingestion of a small amount can be fatal for dogs and cats [46,70], and the pet owners should be appropriately educated.

Opioids were another type of high-risk medications reported in the literature. All the reported opioids in the study were not stored on a counter, suggesting that the study households were able to alleviate the risk of opioid poisoning and diversion to some degree. Locked spaces would be the optimal storage locations for opioids, but the survey did not assess whether the reported opioids were stored in locked spaces. Unlike the previous surveys with at least 30% of their samples having leftover opioids [37,38,39,40,41,42,43,71], the current study only had a small number of opioids reported. The discrepancy could also have been caused by inaccurate reporting or social desirability bias of the sample. The study sample might not have included a reasonable number of households with opioids and UUE medications in general.

#### 4.2.2. Environmental Risk

Almost a half of the reported medications had a Persistence, Bioaccumulation, Toxicity (PBT) score of 4 or higher, where a higher score indicated a higher environmental risk. In contrast, those with insignificant or low toxic risk to the aquatic environment combined took up a similar proportion. This finding highlights that medications without direct toxic effects on the aquatic environment can harm the environment via other mechanisms such as high persistence (P) and bioaccumulation (B). Despite the various ways of pharmaceuticals harming the environment, the literature found that up to 80% of the US patients are not educated about proper disposal methods of medications [11,37,38,39,42]. Additionally, the current disposal mechanisms and systems in the US have apparent limitations. The Resource Conservation and Recovery Act (RCRA) governs the framework for the generation, transportation, treatment, storage, and disposal of hazardous waste, but it recognizes only a small fraction of OTC medications as hazardous waste [52]. A new rule passed under RCRA in 2018 also set the threshold for pharmaceutical waste from healthcare facilities [72]. However, the rule seems to request the stakeholders to accomplish the set outcomes without providing sufficient support for achieving those goals. More efficient support can only be provided after more thoughtful consideration of the sources of pharmaceutical waste.

In addition to addressing the sources of the waste, the environmental effects of pharmaceutical substances need to be more extensively researched. In the current study, more than a half of the reported medications did not have any or enough environmental information available to determine their toxic risk to aquatic organisms or PBT score. The Stockholm report is the most comprehensive resource for the environmental effects of medications to this date, but it lacks considerable evidence and cannot be generalized to countries other than Sweden. PharmEcovigilance is a dimension of pharmacovigilance that governs the environmental effects of pharmaceuticals. The concept of pharmEcovigilance should be more actively promoted for accurate assessment of potential environmental risk and development of interventions protecting the environment from the potential harm [22]. Under this agenda, more pharmaceutical manufacturers should also be encouraged to research the environmental effects of their medications and share the findings with the public.

### 4.3. Objective #3: To Calculate the Potential Cost of the Unused Medications or Medications Reported to Be “Not Taken” and Stored in the US Households

The cost of the unused medications estimated based on the nationally representative sample was extrapolated to a national level, and the result was unremarkable. The national estimate reported by Law et al., was much higher than the estimate from the current study, ranging from $2.4B to $5.4B [13]. Their calculation may have overestimated the cost, as their data from the convenience samples were collected at drug-take-back events. At the same time, their estimate may be more accurate than the estimate of the current study, as they were able to tally the number of units and exact strengths of unused, unwanted and expired (UUE) medications collected from the sample. In spite of the deviation from the published estimate, the basic cost analysis of the current study would promote discussions about potential costs of UUE medications in the US.

Besides the apparent costs of the UUE pharmaceutical products, their invisible costs are equally concerning. When medications are stored at home, the transfer of medication inventories from suppliers to consumers incurs costs for acquisition and storage [47,48]. The limited access to healthcare in the US adds an additional cost to acquisition for most prescription medications. As for the storage costs, solid dosage medications may not take up a huge volume or require significant storage costs. However, liquid formulations such as insulin or biologics may require delicate storage conditions and additional storage costs. The storage costs can be further increased indirectly, considering the risk of harm via intentional or accidental poisoning or drug diversion, and its potential contribution to the total healthcare costs.

### 4.4. Potential Solutions for Risk Mitigation

Most existing interventions such as drug-take-back programs intend to minimize the environmental and poisoning risks by removing the unnecessary stocks stored at home. Their benefits have been studied mostly from an environmental perspective. Although any consolidated data regarding disposal methods of pharmaceutical waste in the US could not be identified during the literature review, most of the collected medications are suspected to be incinerated and contribute to more pollution [73]. On the other hand, medication reuse or drug repository programs may be a more environmentally friendly and economical alternative. As of 2018, 38 states and Guan in the US have enacted laws for medication donation and reuse, but about a third of them still do not have operational programs. In order for medications to be donated, they have to meet multiple criteria including but not limited to being unexpired and unopened in their original, sealed, tamper-evident packaging, and having no signs of adulteration or misbranding. With the stringent provisions, the types of donors and medications are limited to certain oral medications [74,75]. These provisions are necessary as aligned with the general public concerns and perception about medication reuse [23,24,26,76], but innovative approaches such as packaging for pharmaceuticals, enhancing the quality and safety of medications and enabling their reuse are needed [77,78].

Despite the challenges, the medication reuse programs in the US have shown prospects for growth and benefit. Iowa and Wyoming reported their success in redistributing $17.7 million and $12.5 million worth of medications in one fiscal year, respectively [74]. The American Society of Clinical Oncology also publicly expressed its commitment to supporting drug repository programs in 2020. Although their support is only for redistribution of oral medications maintained in a controlled and supervised healthcare environment, this may indicate that more sectors within healthcare are recognizing the need for such programs [75]. In addition, better success and expansion of the repository programs can be realized with services or technologies that streamline donation, and inspection of donated medications. For instance, SIRUM, a non-profit organization in California, which provides streamlined donation packaging and shipping services, has now expanded into Colorado, Oregon, and Ohio [79,80].

Ruhoy et al., however, have determined that these “downstream” approaches may incur high costs and have inefficiently captured all medications accumulated as waste historically [12]. Instead, “upstream” approaches targeting the sources of pharmaceutical waste that can reduce the overall healthcare costs and burden of proper medication disposal should also be considered. Some of the recommended upstream approaches are unit packaging, providing trial scripts for new medications, low-quantity packaging of OTC medications, free samples, and drug repository programs that accept donations from patients [12]. Sweden has developed “Kloka Listan” or the Wise List that provides healthcare clinicians with a list of medications for common diseases recommended based on cost-effectiveness and environmental effects [53]. This type of comprehensive database would greatly help US health providers make more economical and environmentally appropriate decisions when prescribing.

### 4.5. Limitations

The findings suggested certain areas of improvement in healthcare and aspects for which patients and their household members should be better educated. As a household survey, however, the analysis did not reflect the medication use and storage by individuals. Some of the variables could have been more accurately and precisely collected. Both frequencies of use and storage locations did not specify the response collected as “other.” The unit for quantities and strengths of medications, and the type of pets owned by the households were not specified as well. Sampling bias, recall bias and social desirability bias may have led to under-reporting of certain medications. Without collecting actual poisoning incidences and disposal methods, the results of the risk analyses could not be determinative. The Stockholm report that was utilized as the reference for the environmental risk analysis did not have conclusive evidence for various medications. Their Sweden-based data also may not be completely applicable in the US.

In-person and on-site assessment would be the most accurate method for evaluating medications stored at home and overcome the limitations that the current study had. The study by Sorenson et al. that found the association between the number of medications stored at home and the risk factors and health outcomes was done through in-person home visits in Australia [28]. Similar direct observations of the medication use, storage, and disposal by investigators in US homes may help tailor patient education and systemic interventions to minimize waste and maximize the efficiency of care and medication use.

## 5. Conclusions

Various areas of medications stored at home including, the use, storage, and poisoning and environmental risk, have been discussed in this paper. The study especially highlighted the negative implications of medications stored in US households. Notably, a significant portion of the medications stored in the participating households could put the vulnerable populations at risk of accidental exposure and harm the environment. Without studying more about these risks and their intricate associations with patients and household members, the society may keep suffering from the negative consequences. Thus, the findings attest to the dire need for more extensive research in this field to complements the limitations of the study. Those limitations include, but are not limited to, a small sample size and the explorative nature of the study that could not measure direct risks. Such research will guide efficient development and implementation of innovative interventions like medication reuse to prevent any potential harm.

## Figures and Tables

**Table 1:** Number of medications stored by households with at least one resident older than 65 years vs. without a resident older than 65 years.

Number of Medications Stored in Households	Number of Househods Storing at Least One Medication (*n* = 154)		*p*-Value ^a^
	With at Least One Resident Older than 65 Years (*n* = 46)	Without a Resident Older than 65 Years (*n* = 108)	
1–4 medication(s)	102 (66.2%)		0.10
	27 (58.7%)	75 (69.4%)	
5–10 medications	42 (27.3%)		
	13 (28.3%)	29 (26.9%)	
>10 medications	10 (6.5%)		
	6 (13%)	4 (3.7%)	

^a^ Fisher’s exact test.

**Table 2:** Indications of the medications stored in the households (*n* = 404 ^a^).

Prescription Medications				OTC Medications	
Non-Controlled		Controlled Substances			
Indications	*n* = 236	Indications	*n* = 25	Indications	*n* = 143
Cardiovascular therapy	79 (33.5%)	Mental health ^c^	15 (60%)	Pain	53 (37.1%)
Mental health	44 (18.6%)	Pain ^d^	9 (36%)	Supplements	26 (18.2%)
Endocrine therapy	39 (16.5%)	Weight loss	1 (4%)	Gastrointestinal therapy	19 (13.3%)
Antibiotics	9 (3.8%)			Cardiovascular therapy and pain	11 (7.7%)
Others ^b^	57 (24.1%)			Others ^e^	34 (23.8%)

^a^ “Invalid” and “duplicate” responses were excluded from the current analysis, ^b^ Includes indications with counts of 8 or fewer (complete counts provided in Appendix F), ^c^ Notably includes 9 benzodiazepines and 1 non-benzodiazepine hypnotic sedative, ^d^ Notably includes 4 opioids and 1 neuropathic pain, ^e^ Includes indications with counts of 8 or fewer (complete counts provided in Appendix F).

**Table 3:** Medication frequency of use.

Frequency of Use (*n* = 465)	
Taken daily	306 (65.8%)
Taken as needed	130 (28%)
Not taken, saving for future	12 (2.6%)
Not taken, would like to discard	7 (1.5%)
Other	10 (2.2%)

## Data Availability

Data files are stored in encrypted format at University of Minnesota. Requests for access to the files may sent to the corresponding author at schom010@umn.edu.

## Appendix A. Process of Assigning Medication Indications

The categorization of indications intended to be as inclusive as possible without having much overlap among the indications. For instance, “cardiovascular therapy medications” included antihypertensive, anticholesteremic, antithrombotic, antianginal, and heartrate control medications. For medications with multiple active ingredients, their most likely common indication was assigned. For instance, the common indication of a combination of acetaminophen, dextromethorphan, and doxylamine was “cold,” rather than a more specific indication for each ingredient. If the multiple active ingredients, however, did not have a common indication, all of their typical indications were assigned. For example, the indication of Yosprala containing aspirin and omeprazole was categorized as “cardiovascular therapy/gastrointestinal therapy.” If a medication had multiple indications, the more commonly used indication was reported. For example, gabapentin was initially developed to treat seizures, but in current practice, it is predominantly used for neuropathic pain. Hence, for this study, its indication was categorized as “neuropathic pain.” If one medication had multiple competing indications equally common in practice, then all of the indications were reported. For example, hydroxyzine was categorized as “mental health therapy” for its use for both anxiety and “allergies.” For medications with various indications and without any distinct, predominantly common indications, their medication class was used for categorization. For instance, methotrexate which can be used for various autoimmune diseases was categorized as “immunosuppressants.” For responses classified as “duplicate,” when a participant reported a medication in its brand name first and generic name afterward, for instance, Advil and ibuprofen, only the first response was assigned with an indication, and the rest was deemed “duplicate.”

## Appendix B. Crude Responses (Prescription-only Non-Controlled Medications)

**Indications**	**Entries**
Cardiovascular therapy	lipitor, lipitor, Simvastatin, provastatin, AMLODIPINE, lisinopril, atenolol, lisinopril, lisinopril, linsinopril, Simvistatin, simvastatin, Benazapril, elanapril, Benezipril, lisinopril, rosuvastatin calcium, carvedilol, hydrocholotyide, lisinopril, Olmesartan Medoxomil, Lisinoprill, Diltiazem, Verapamil, Pravastatin, amlodipine, Spironolactone, metoprolol tarrate, metoprolol l tartrate, amlodopin, atorvastatin, losartan, diltiazem, lisinopril, Nifedipine, simvastatin, Lisinopril, atenolol, pravostatin, carvedilol, fenofibrate, Propranolol, Trilipix, warfarin, eliquis, hydrochlothazide, Losartan Potassium, simvastatin, Lovastatin, finofibrate, lisinopril, Simvastatin, Isosorbide Mononitrate, ATORVASTATIN, Atorvastatin, Brillintal, losartan, lisinopril, pravastatin, propranolol, clopidogrel, LOSARTAN, Diltiazem, lisinopril, Astrovastatin, Losartan, sotalol, Metoprolol Tartrate, Metroprolol Tartrate, metoprolol, spironalactone, clopidogrel, pravastatin, isosorbide mononitrate, niacin, metoprolol succ er, metoprolol, lovastatin, lisinopril
Mental health	CYMBALTA, Zoloft, Duloxetine, Paxel, lexapor, zoloft, lexapro, Fluoxetine, abilify, paroxetine, Paxil, Prozac, Paxil, ESCITALOPRAM, Sertraline, Paxil, celexa, Risperidone, Geodone, citaopram, paxil, Prozac, duloxetine, Lithium, Risperidone, Lamotrigine, Zoloft, Wellbutrin, Wellbutrin, cymbalta, aripiprazole, Buspirone, cymbolta, effexor, prozac, duloxetine, Atomoxetine HCL, duloxetine, escitalopram oxalate, venaflaxine, Buspirone, buspar, QUETIAPINE, Buspirone
Endocrine therapy	metformin, Metformin, Levothyroxin, levothyroxine, MEDFORMIN, Fosamax, Glimepride, levoxyl, Levoxylthrine, metformin, Starlix, levothyroxine sodium, allopernol, levothyroxine, Levothyroxine, Alendronate, Tradjenta, synthroid, Metformin, Finesteride, metformin, Calcitriol, levthyroine, Glimepiride, Onglyza, metformin, glimeperide, glipizide, glipizide, prednisone, Lantus, lantus, humalog, apidra, Victoza, Estrofem, vivelle dot patch, estarylla, Microgestin
Antibiotics	Amoxicillin, zythromician, amoxicillin, CLINDAMYCIN, ciprofloxacin, metronidazole, Peniclin, penacillian, doxycycline
Muscle spasm	tizanadine, tizanidine, cyclobednzaprine, cyclobenzaprine hcl, cyclobenzaprine, Tizanidine, cyclobenzapran, tizanidine
Insomnia	trazadone, Trazadone, Mirtazapine, Mirtazapine, trazodone, remeron, mirtazapine
Inhalers (COPD, Asthma)	Ventolin inhaler, flovent 220, proair inhaler, ventolin, proair albuterol, advair
Neuropathic pain	gabapentin, gabapentin, Gabapentin, gabapentin, gabapentin, GABAPENTIN
Specialty injections	humira, humira, Remicade, humira, humira, enbrel
Anticonvulsant	dilantin, zonegran, zonisamide, CARBAMAZEPINE, carbamazepine
Gastrointestinal therapy	bentyl, librax, dexilant, dicyclomine
Fluid retention	furosemide, furosemide, furosamide
Pain	meloxicam, meloxicam, Meloxicam
Asthma (oral)	singular, singular
Cardiovascular therapy and mental health	Clonidine HCl, Clonidine
Incontinence	Vesicare, oxybutynin
Immunosuppressants	methotrexate, ARAVIA
Mental health and allergies	hydroyoxyzine
Anticonvulsant and antiglaucoma	acetazolamide
Cough	BENZONATATE
Cardiovascular therapy and gastrointestinal therapy	yosorala
Hair loss (topical)	vaniqa
Antiviral (HIV)	atripla
Migraine	immetrex
Steroid (topical)	Triamcinalone
Electrolyte supplementation	klor con

## Appendix C. Crude Responses (Controlled Substances)

**Indications**	**Entries**
Mental health	Phenobarbiyol, Concerta, ritalin, Adderall, Focalin *Benzodiazepines* clonazepam, Xanax, ativan, Xanax, Klonopin, Xanax, xnax, lorazepam, alprazolam *benzodiazepine-like non-benzodiazepines* Ambean
Pain/controlled	tramadol, Tramadol, Tramadol, tramadol hcl *Opioids* oxycodone, vicodin, Norco, Norco *Neuropathic pain* lyrica
Weight loss/controlled	phentermine

## Appendix D. Crude Responses (OTC Medications)

**Indications**	**Entries**
Pain	Tylenol, alieve, advil, tylenol, advil, ibuprofen, advil, ADVIL, aleeve, Acetametophin, advil, Ibuprofen, advil, Acetaminophen, advil, aleve, Advil, ibprofen, Advil, ibupfrofen, Tylenol, tylenol, advil, tylenol, tylenol, aleve, acetaminophen, tynol, motrin, tylanol, tylenol, Ibrfrophen, advil, Tylenol, advil, Tylenol, ibuprofen, IBUPROFEN, tylenol, Ibiprogen, ibuprofen, advil, Ibuprofen, NAPROXEN, tylenol, Extra Strength Tylenol, IBUPROFEN, TYLENOL, acetaminophen, Ibruprofen pm, Naproxen, ibuprofen, ibuprofen
Supplements	pnv, vitamins, multivitamins, b12, Flintstone Vitamins, folic acid, oneaday, cinnamon, iron, cholecalciferol vd3, calcium with D, Vitamin C, Biotin, vitamin d, multi-vitamin, b12, B12, cinnamon, ONE DAY WOME;S MULTIVITAMINS, vitamin d, IRON, coq10, vitamin d3, glucosamine, magnesium, hydrangea root
Gastrointestinal therapy	omezaprole, omeprezole, omeprosole, SENNA-LAX, Zantac, Omeprazole, Equate antacid, omeprazole, meta-mucil, omeprazol, Omeprazole, OMEPRAZOLE, nexium, omeprazole, Pepto Bismal, Omeprazole, senexon, polyethylene glycol, simethicone
Cardiovascular therapy and pain	aspirin, ASPHRAN, aspirin, aspirin, ASPIRIN, Aspirin, aspirin, aspirin, aspirin, aspirin, aspirine
Allergies	zyrtec, Loratadin, claritin, Fexofenadine, allegra, wal-zyr, loratadine, Xyzal
Cold	NyQuil, Advil PM, dimatep, Tylenol PM, nyquil, acetaminophen phenylephrine dextromethorphan, dextromethorphan doxylamine succinate
Nasal sprays (decongestants)	nasacort, Flonase, flonase, flonase, flournase, luticasone
Allergies and insomnia	Benadryl, Simply Sleep, benadryl
Cardiovascular therapy	fish oil, fish oil
Migraine	excedrin, Excedrin
Pain (topical)	arnicare, Therapain
Eye drops	Refresh
Insomnia	Melatonin
Sore throat (topical)	Chloraseptic
Antiseptic (topical)	hydrogen peroxide

## Appendix E. “Duplicate” and “Invalid” Responses

Duplicate	advil, ibuprophen, levoxyl, advil, atorvastatin, advil, Lipitor, VITAMINS
Invalid	good, ahn, one, yes, one, one, Nore, hgygu, borg, medizel, Fevers, gius, metrolmsop, gtreth, one, as, sustatin, unknown, dol, idk, CAPSULES, Jetson, BANDAGE, Fevers, oxy, metrokoloious, Unsure, Muscle Relax, birth control, trats, Nite Time, ear drops, Sleep Aid, Exelium, Bayer, tyroid, after sun lotion, Avien, callous liquid, mucus relief, Anti Allergy, birth control, Sinus Relief, hydrochloride, allergy relief

## Appendix F. Complete Counts of Indications of Medications Stored in the Households (*N* = 404)

**Prescription Medications**				**OTC Medications**	
**Non-Controlled**		**Controlled Substances**			
**Indications**	***N* = 236**	**Indications**	***N* = 25**	**Indications**	***N* = 143**
Cardiovascular therapy	79 (33.5%)	Mental health	15 (60%)	Pain	53 (37.1%)
Mental health	44 (18.6%)	Pain	9 (36%)	Supplements	26 (18.2%)
Endocrine therapy	39 (16.5%)	Weight loss	1 (4%)	Gastrointestinal therapy	19 (13.3%)
Antibiotics	9 (3.8%)			Cardiovascular therapy and pain	11 (7.7%)
Muscle spasm	8 (3.4%)			Allergies	8 (5.6%)
Insomnia	7 (3.0%)			Cold	7 (4.9%)
Inhalers (COPD, Asthma)	6 (2.5%)			Nasal sprays (decongestants)	6 (4.2%)
Neuropathic pain	6 (2.5%)			Allergies and insomnia	3 (2.1%)
Specialty injections	6 (2.5%)			Cardiovascular therapy	2 (1.4%)
Anticonvulsant	5 (2.1%)			Migraine	2 (1.4%)
Gastrointestinal therapy	4 (1.7%)			Pain (topical)	2 (1.4%)
Muscle spasm	8 (3.4%)			Eye drops	1 (0.7%)
Insomnia	7 (3.0%)			Insomnia	1 (0.7%)
Inhalers (COPD, Asthma)	6 (2.5%)			Sore throat (topical)	1 (0.7%)
Neuropathic pain	6 (2.5%)			Antiseptic (topical)	1 (0.7%)
Specialty injections	6 (2.5%)				
Anticonvulsant	5 (2.1%)				
Gastrointestinal therapy	4 (1.7%)				
Fluid retention	3 (1.3%)				
Pain	3 (1.3%)				
Asthma (oral)	2 (0.9%)				
Cardiovascular therapy and mental health	2 (0.9%)				
Incontinence	2 (0.9%)				
Immunosuppressants	2 (0.9%)				
Mental health and allergies	1 (0.4%)				
Anticonvulsant and antiglaucoma	1 (0.4%)				
Cough	1 (0.4%)				
Cardiovascular therapy and gastrointestinal therapy	1 (0.4%)				
Hair loss (topical)	1 (0.4%)				
Antiviral (HIV)	1 (0.4%)				
Migraine	1 (0.4%)				
Steroid (topical)	1 (0.4%)				
Electrolyte supplementation	1 (0.4%)				
